# Spatially-Aware Reliability Modeling for BEV LiDAR 3D Vehicle Detection

**DOI:** 10.3390/s26134229

**Published:** 2026-07-03

**Authors:** Nanzhou Hu, Zhe Zhang, Dayong Wu, Bahar Dadashova, Fikriyah Winata, Anthony M. Filippi

**Affiliations:** 1 Department of Geography, Texas A&M University, College Station, TX 77843, USA; nz972019@tamu.edu (N.H.); winata@tamu.edu (F.W.); filippi@tamu.edu (A.M.F.); 2 Texas A&M Transportation Institute, College Station, TX 77843, USA; j-wu@tti.tamu.edu (D.W.); b-dadashova@tti.tamu.edu (B.D.)

**Keywords:** LiDAR sensing, bird’s-eye-view (BEV) detection, 3D object detection, localization reliability, quality calibration, score calibration

## Abstract

Bird’s-eye-view (BEV) LiDAR detectors provide an efficient representation for real-time 3D perception, but their confidence scores may be imperfectly aligned with the localization quality of decoded 3D boxes. This score–localization mismatch can reduce the reliability of detection ranking, especially under strict IoU criteria and range-dependent LiDAR observations. To address this issue, we propose Spatially-Aware Quality Calibration (SAQC), a lightweight reliability-oriented scoring framework for BEV LiDAR 3D vehicle detection. SAQC estimates localization quality from coordinate-augmented local BEV feature patches around detected object centers, fuses the estimated quality with the raw detector score for quality-aware ranking, and applies post hoc sigmoid calibration with soft IoU targets for numerical quality alignment. Experiments on the KITTI Car validation split using an SFA3D-style center-based detector show that SAQC improves Moderate 3D AP from 89.13 to 89.57 at IoU = 0.7 and from 74.40 to 75.72 at IoU = 0.8. It also increases score–IoU Spearman correlation from 0.3685 to 0.4043 and reduces post-calibration Q-ECE from 0.0284 to 0.0183, while maintaining 103.6 FPS. These results indicate that local BEV spatial context can improve score–localization reliability without modifying decoded box geometry.

## 1. Introduction

Three-dimensional perception is an essential capability for LiDAR-based sensing systems in autonomous driving, mobile robotics, intelligent transportation systems, and infrastructure monitoring [1,2,3]. In these applications, perception systems are used to detect objects and to provide spatial evidence for downstream tracking, planning, risk assessment, and decision-making [4,5]. LiDAR (Light Detection and Ranging) is particularly useful and widely used in this context because it directly measures the three-dimensional geometry of outdoor scenes and is less sensitive to illumination changes and appearance variations than image-based sensing [6,7]. With the rapid development of deep learning, LiDAR-based object detection has made substantial progress, enabling efficient object-level 3D sensing in real-world environments [8].

Among existing LiDAR representations, bird’s-eye view (BEV) has emerged as one of the most effective interfaces for 3D perception. By projecting point clouds onto a metric-preserving horizontal plane, BEV retains the spatial structure needed for localization while enabling efficient convolution-based inference. Earlier voxel-based and pillar-based detectors demonstrated that structured BEV encoding can achieve a favorable balance between accuracy and computational efficiency [9,10], and later methods further improved BEV-based perception through richer feature aggregation and stronger geometric reasoning [11,12,13]. As a result, BEV-based LiDAR detection has become a widely adopted paradigm for efficient 3D perception.

Although these studies substantially improved box prediction, a related question remains less directly examined in BEV LiDAR detection: whether the final detector score reliably reflects the localization quality of the decoded box. In many scenarios, detector scores are used as the primary basis for ranking, filtering, and retaining candidate objects. High-scoring detections are often treated as more dependable spatial evidence in downstream pipelines [14]. However, classification confidence and box regression quality are not optimized in the same way, so a detection may still receive a high score even when its localization quality is unsatisfactory [15,16,17]. As a result, detector scores can remain useful for object presence estimation while being imperfectly aligned with the geometric localization quality. In the BEV LiDAR setting, this mismatch is particularly important because downstream use often depends on whether high-ranked detections are also well localized in metric space.

Existing studies have addressed related aspects of this problem from two main directions. First, quality-aware scoring methods showed that classification confidence should not be treated as equivalent to localization quality and that explicit quality estimation can improve detection ranking [15,16,18,19]. Second, calibration studies have indicated that detector scores are often numerically misaligned with empirical correctness or quality and that post hoc correction can provide useful reliability gains [20,21,22]. These studies provide an important foundation, but they do not fully address a question that is especially relevant in the BEV LiDAR setting: can localization reliability itself be modeled as a spatially grounded property of local BEV structure?

This question matters because the reliability of BEV detection is not determined solely by the feature response at a single center location. In many cases, whether a predicted box is well aligned depends on the surrounding local spatial neighborhood, including occupancy patterns, object extent, and nearby spatial arrangement. In this sense, localization reliability in BEV detection is not only a scoring problem, but also a local spatial modeling problem. From a LiDAR sensing perspective, this distinction is important because detections are often used as object-level spatial measurements rather than as isolated classification outputs. A reliability estimate that better reflects local geometric support may therefore improve the practical usefulness and interpretability of detector scores in downstream perception, tracking, and decision-making pipelines.

**Figure 1:**
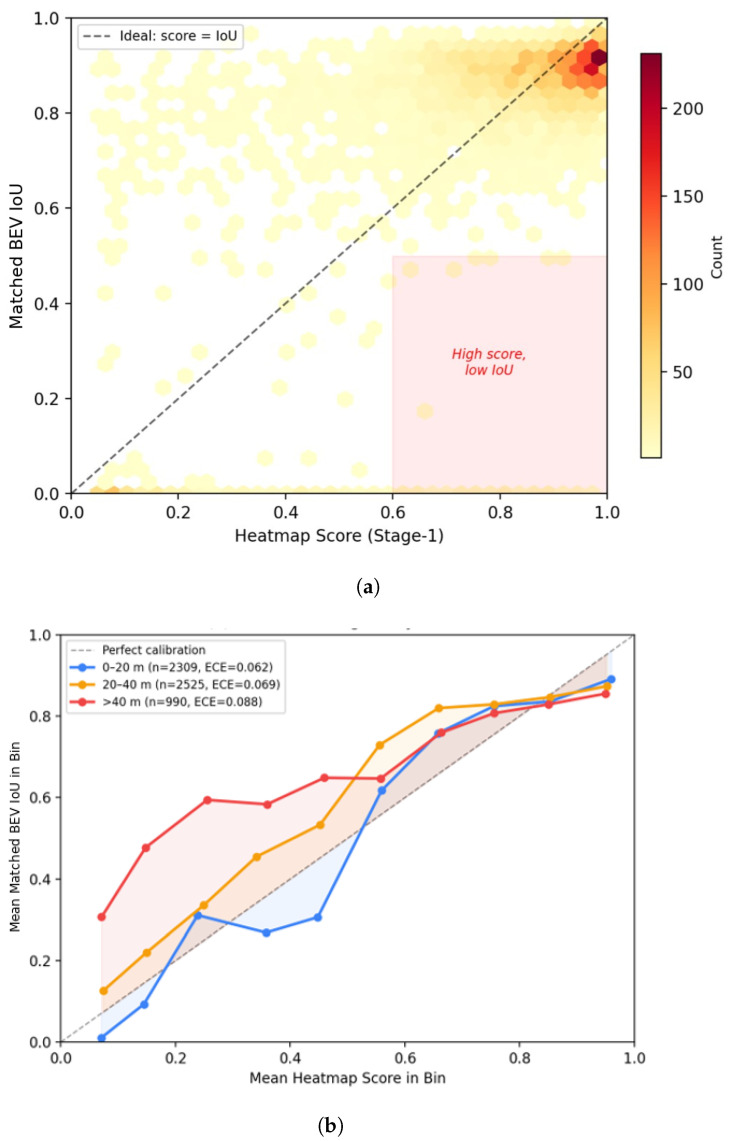
Motivation of the score–localization reliability problem in BEV LiDAR 3D detection. (**a**) The joint distribution of raw detector score and matched BEV IoU for validation detections shows that some high-score predictions still have unsatisfactory localization quality. (**b**) Distance-wise score–quality reliability curves show that the mismatch between score and matched localization quality becomes more pronounced at longer sensing ranges.

In this study, spatial reliability is used as an operational description of score–localization reliability in BEV space. A detection is considered more spatially reliable when its score is supported not only by the center heatmap response, but also by local BEV evidence around the decoded box, such as occupancy structure and vehicle footprint. For example, two detections may have similar heatmap scores, but the one whose box better matches the local BEV structure should receive a score more consistent with its localization quality. This score–localization mismatch can be observed directly in the baseline detector outputs. Figure 1 illustrates the relationship between the raw detector score and the matched BEV IoU on the KITTI Car validation split. Although high-quality detections are generally concentrated in the high-score region, a non-negligible number of predictions still receive high raw scores despite having relatively low localization quality. The distance-wise reliability curves further indicate that this mismatch becomes more pronounced at longer sensing ranges, where LiDAR observations are usually sparser. These observations motivate the proposed SAQC framework, which estimates localization quality from local BEV spatial evidence rather than relying only on the center heatmap score.

To address this issue, we propose a Spatially-Aware Quality Calibration (SAQC) framework, a reliability-oriented scoring framework for BEV LiDAR 3D vehicle detection. The framework introduces a lightweight Local Spatial Quality Head (LSQH) that estimates localization quality from local BEV feature patches centered on detected objects. The predicted quality is then fused with the raw detector score to produce a more quality-aware ranking score, followed by a final post hoc sigmoid calibration step to improve score calibration. Importantly, the proposed method does not introduce an explicit box-refinement step. Instead, it operates at the scoring stage and is designed to make detector scores more informative about localization quality while keeping the base detector architecture lightweight. This scope is distinct from physical LiDAR signal-propagation modeling. The proposed framework does not estimate atmospheric extinction, correct range measurements, or simulate adverse-weather degradation. Rather, it addresses the downstream reliability of detector outputs after the point cloud has already been encoded and decoded by a BEV detector.

More specifically, this study addresses the score–localization reliability problem in BEV LiDAR 3D vehicle detection. Given a set of decoded detections produced by a fixed BEV detector, each detection has a predicted 3D box, a raw detector score, and local BEV feature evidence around its predicted center. The problem considered here is how to transform the final detection score so that higher-ranked detections are more likely to correspond to better localized boxes, while the decoded 3D box geometry remains unchanged. Therefore, the proposed method should be interpreted as a reliability-oriented scoring layer rather than as a LiDAR ranging correction method, a geometric box refinement module, or an adverse-weather propagation model.

The scientific novelty of SAQC lies in treating localization reliability in BEV LiDAR detection as a local spatial inference problem. Existing quality-aware scoring methods primarily estimate localization quality as an auxiliary scalar signal, whereas SAQC explicitly uses coordinate-augmented local BEV feature neighborhoods to infer whether the decoded box is spatially well supported. The method further separates ranking-oriented quality fusion from post hoc numerical calibration, allowing the score to be evaluated both as a ranking signal and as a calibrated estimate of localization quality.

The main contributions of this study are as follows:

1. We formalize BEV LiDAR score reliability as a constrained multicriteria scoring formulation in which decoded box geometry is fixed, and the scoring layer is designed and evaluated with respect to localization-quality ranking, calibration, and real-time feasibility.
2. We propose a Local Spatial Quality Head that estimates localization quality from coordinate-augmented local BEV feature patches, thereby modeling localization reliability as a spatially structured property rather than as center-point confidence alone.
3. We show on the KITTI Car validation split that SAQC improves strict-IoU 3D AP, increases score–IoU monotonicity, reduces calibrated quality error, and maintains real-time inference speed. These results indicate that the benefit comes mainly from improved score ordering and calibration rather than from box-coordinate refinement.

The remainder of this paper continues as follows. Section 2 reviews related work on BEV-based LiDAR 3D object detection, quality-aware scoring, and detector calibration. Section 3 presents the proposed framework, including the Local Spatial Quality Head, score fusion, and post hoc calibration strategy. Section 4 reports the experimental setup and results. Section 5 discusses the implications and limitations of the proposed method. Section 6 concludes the paper.

## 2. Related Work

This section reviews prior studies related to the proposed reliability-oriented scoring framework. We first summarize BEV-based LiDAR 3D object detection methods, which provide the detection architectures and feature representations relevant to this study. We then review quality-aware scoring and localization-quality estimation methods that address the mismatch between classification confidence and localization quality. Finally, we discuss detector reliability and calibration studies, which motivate the post hoc quality calibration component of SAQC.

### 2.1. BEV-Based LiDAR 3D Object Detection

LiDAR-based 3D object detection has developed from early voxelized and pillar-based representations to more expressive point-voxel and center-based BEV paradigms. Early studies such as VoxelNet, SECOND, and PointPillars demonstrated that structured representations of point clouds can support efficient and accurate 3D object detection [9,10,23]. Subsequent methods, including PointRCNN, Part-A^2^ Net, PV-RCNN, and CenterPoint, further improved proposal generation, feature aggregation, and geometric reasoning in point-cloud detection [11,12,24,25]. More recently, BEV has also become an important interface for efficient perception and multi-sensor fusion, as shown by BEVFusion and related approaches [13].

These studies have substantially improved representation learning, localization accuracy, and computational efficiency in LiDAR perception. However, most existing BEV-based detectors primarily focus on generating better boxes rather than on whether the final detector score reliably reflects the geometric quality of the predicted box. In practice, this distinction is important because highly ranked detections are often treated as the most trustworthy outputs in downstream perception, tracking, and decision-making pipelines. Therefore, the present work focuses not on box refinement but on improving the relationship between detector scores and localization quality in BEV LiDAR detection. More specifically, the proposed SAQC framework differs from these prior quality-aware scoring methods in three aspects. First, the quality signal is inferred from a coordinate-augmented local BEV feature neighborhood rather than from a point-wise feature or a global scalar branch alone. Second, the predicted quality is used as a ranking-oriented spatial reliability signal before a separate post hoc calibration step, so the method distinguishes score ordering from numerical score calibration. Third, the method is evaluated in a BEV LiDAR 3D vehicle detection setting, where localization quality depends on local occupancy structure, vehicle footprint, and spatial alignment in metric space. Therefore, SAQC should be interpreted as a spatially informed reliability layer for BEV LiDAR detection rather than as a direct replacement for general quality-aware 2D detection methods.

### 2.2. Quality-Aware Scoring and Localization-Quality Estimation

The mismatch between classification confidence and localization quality has been widely recognized in object detection. IoU-Net was among the earliest studies to explicitly predict localization quality and use it to improve detection and non-maximum suppression [15]. Later studies further emphasized that classification confidence alone is insufficient for ranking detection quality. Generalized Focal Loss incorporated localization quality into a classification-style learning framework. At the same time, related approaches such as IoU-aware dense detection, VarifocalNet, and Generalized Focal Loss V2 continued to show that explicit quality estimation can improve ranking consistency and detection performance [16,18,26,27]. More recent work has also revisited the score–IoU relationship in quality-aware detection from the perspective of more reliable ranking and localization-quality prediction [19]. Quality-aware confidence estimation has also been studied in LiDAR-based 3D object detection. 3D IoU-Net introduced a 3D IoU prediction branch to estimate detection confidence for point-cloud 3D object detection, while CIA-SSD used IoU-aware confidence rectification to reduce the mismatch between classification confidence and localization accuracy [28,29]. More recently, LidarMetaDetect proposed a lightweight post-processing scheme for prediction quality estimation in LiDAR object detection [30]. These studies are closely related to the present work because they also recognize that native detector confidence may not adequately represent localization quality. However, SAQC differs from these prior directions by estimating localization reliability from coordinate-augmented local BEV feature neighborhoods and by separating ranking-oriented score fusion from post hoc numerical calibration.

Together, these studies established that detector confidence should not be equated with localization quality. However, most of the existing work treated localization quality mainly as an auxiliary scoring signal, and many representative methods were developed in two-dimensional detection settings. By contrast, the present study asks a more specific question in the BEV LiDAR setting: whether local BEV spatial context itself can provide explicit evidence for localization-quality estimation. In BEV detection, the quality of a decoded box depends not only on the detector response at a single location but also on the spatial neighborhood around it, including local occupancy structure, object extent, and the surrounding spatial arrangement. Therefore, our work builds on quality-aware scoring but reframes localization-quality estimation as a local spatial inference problem in BEV LiDAR detection.

### 2.3. Reliability and Calibration of Object Detectors

Beyond localization-aware scoring, recent research has also highlighted broader issues in detector reliability and confidence calibration. Guo et al. [20] showed that modern neural networks are often poorly calibrated, and that simple post hoc methods, such as temperature scaling, can still be effective. For object detection, subsequent studies extended calibration analysis beyond class confidence by considering additional spatial and structural factors, including box location, scale, and multivariate prediction structure [21,31,32]. Other work explored train-time calibration objectives and uncertainty-aware detection, further emphasizing that reliability should be treated as a substantive problem rather than a secondary evaluation detail [22,33,34].

However, calibration-oriented studies mainly focus on mapping detector outputs to more accurately calibrated confidence values after prediction. In contrast, robustness-oriented LiDAR studies often focus on sparse observations or uncertain inputs [17]. Neither direction explicitly models the relationship between detector score and localization quality through local BEV spatial context. Our work complements calibration-oriented studies by first improving score–quality consistency through local spatial modeling and then applying post hoc calibration to the resulting quality-aware score. In this way, the proposed framework connects quality-aware scoring and detector calibration within a spatially grounded BEV LiDAR setting.

## 3. Methodology

This section presents the proposed SAQC framework from problem formulation to implementation. We first provide an overview of the reliability-oriented scoring pipeline and then define the score–localization consistency problem under the constraint that decoded 3D box geometry remains unchanged. We subsequently describe the Local Spatial Quality Head, the quality-supervision strategy, and the quality-aware score fusion and post hoc calibration procedures.

### 3.1. Framework Overview

This study proposes a Spatially-Aware Quality Calibration (SAQC) framework, a reliability-oriented scoring framework for BEV LiDAR 3D vehicle detection. Unlike conventional quality-aware scoring and calibration methods that treat localization quality as a scalar prediction problem, the proposed framework models localization reliability as a spatially grounded property in BEV space. Specifically, we hypothesize that the reliability of a decoded detection depends on local spatial context, including occupancy structure, object extent, and the surrounding spatial arrangement. This perspective motivates the proposed local BEV-based quality estimation approach.

**Figure 2:**
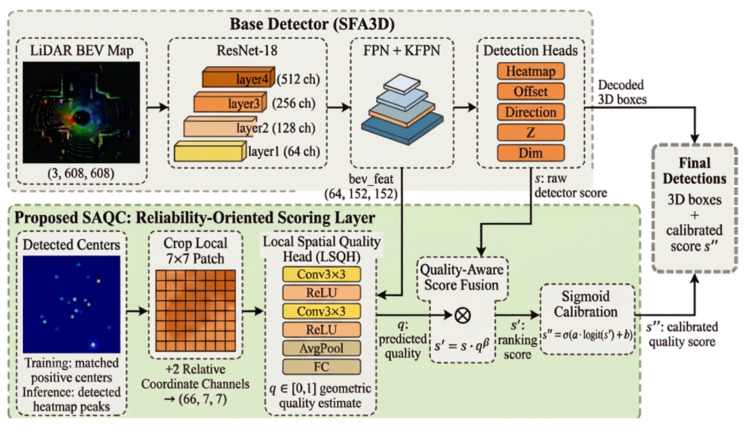
Overview of the proposed Spatially-Aware Quality Calibration (SAQC) framework as a reliability-oriented scoring layer for BEV LiDAR 3D vehicle detection.

As illustrated in Figure 2, the framework consists of two parts: a base BEV detector and a quality estimation branch with a calibration module. The base detector takes a LiDAR BEV map as input and produces decoded 3D bounding boxes together with the corresponding raw detection score *s* [35]. At the same time, an intermediate BEV feature map, denoted as bev_feat, is extracted from the detector backbone–neck pipeline. The BEV feature map preserves local spatial information around detected objects and serves as the feature source for quality estimation.

For each detected center, a local feature patch is cropped from bev_feat and passed to a lightweight Spatial Quality Head (LSQH) to predict a scalar quality score q∈[0,1]. The predicted quality is intended to reflect the localization quality of the decoded detection based on local BEV evidence, rather than to provide an additional class-confidence score. The predicted quality is then fused with the raw detector score to obtain a quality-aware score $s^{\prime }$. Because the fused score is primarily designed for quality-aware ranking, a final post hoc sigmoid calibration step is further applied to produce a calibrated score $s^{\prime \prime }$. In this way, the proposed framework separates two related but distinct objectives: the fusion step improves score structure for ranking. In contrast, the calibration step adjusts the score scale to improve calibration quality. Throughout the whole process, the decoded 3D box geometry remains unchanged. Therefore, the proposed framework serves as a lightweight, reliability-oriented scoring layer on top of the base detector rather than a geometric refinement module. The proposed SAQC framework is designed for center-based BEV LiDAR detectors that provide a dense intermediate BEV feature map aligned with decoded detection centers. Specifically, SAQC assumes that each detection can be associated with a feature-map location $u_{i}$, so that a local k×k BEV feature patch can be sampled around the predicted object center. This assumption is naturally satisfied by heatmap-based BEV detectors, where object centers are decoded from spatial response maps. However, SAQC is not architecture-agnostic in its current form. Point-based, proposal-based, range-view, and anchor-based detectors do not necessarily provide the same dense, center-aligned BEV feature representation. Applying SAQC to these architectures would require redefining how local spatial evidence is sampled and associated with each decoded detection.

The following subsections describe the base detector and the score–quality inconsistency problem, followed by the proposed LSQH, its supervision strategy, and the score fusion and calibration procedure.

### 3.2. Problem Formulation

For a given input frame, let *F* denote the intermediate BEV feature map. Let $d_{i}=\left(b_{i},s_{i},u_{i}\right)$ denote the *i*-th decoded detection produced by the BEV LiDAR detector, where $b_{i}$ is the decoded 3D bounding box, $s_{i}\in \left[0,1\right]$ is the raw detector score, and $u_{i}$ is the corresponding BEV feature-map location. The set of decoded detections is denoted by $D={\left\{d_{i}\right\}}_{i=1}^{M}$. For matched detections, let $y_{i}=I_{\mathrm{BEV}}\left(b_{i},b_{i}^{*}\right)$ denote the localization-quality target, where $b_{i}^{*}$ is the matched ground-truth box. The BEV IoU function $I_{\mathrm{BEV}}\left(\cdot ,\cdot \right)$ is formally defined in Section 3.5. The objective of SAQC is not to introduce an additional box-refinement step, but to transform the final detection score so that it is more consistent with localization quality.

(1) $$s_{i}^{\prime }\left(\theta ,\beta \right)=h_{\theta ,\beta }\left(s_{i},F,u_{i}\right),\;s_{i}^{\prime \prime }\left(\theta ,\beta ,a,b\right)=g_{a,b}\left(s_{i}^{\prime }\left(\theta ,\beta \right)\right),\;{\hat{b}}_{i}=b_{i}.$$

Here, $h_{\theta ,\beta }\left(\cdot \right)$ denotes the quality-aware ranking transformation implemented by local spatial quality estimation and score fusion, while $g_{a,b}\left(\cdot \right)$ denotes the post hoc sigmoid calibration mapping. The fused score $s_{i}^{\prime }$ is used as a ranking-oriented score, whereas the calibrated score $s_{i}^{\prime \prime }$ is used as a numerical estimate of localization quality. The equality ${\hat{b}}_{i}=b_{i}$, where ${\hat{b}}_{i}$ denotes the box associated with the final SAQC-scored detection, emphasizes that the SAQC scoring layer does not alter the decoded box geometry.

Let $r_{i}\left(\theta ,\beta \right)$ denote the scored detection consisting of the fixed box $b_{i}$ and the ranking score $s_{i}^{\prime }\left(\theta ,\beta \right)$, and let $R\left(\theta ,\beta \right)={\left\{r_{i}\left(\theta ,\beta \right)\right\}}_{i=1}^{M}$ denote the corresponding scored-detection set. At the design and evaluation level, the desired scoring layer is assessed by the following constrained multicriteria mapping:

(2) $$\begin{array}{cc} M(\theta ,\beta ,a,b)\;=\;( & {\mathrm{AP}}_{\tau }\left(R(\theta ,\beta ),G\right),\\ & \rho_{S}\left(s^{\prime }\left(\theta ,\beta \right),y\right),\\ & -\mathrm{QECE}\left(s^{\prime \prime }\left(\theta ,\beta ,a,b\right),y\right)),\;s.t.\;{\hat{b}}_{i}=b_{i},\;\Delta t\left(\theta \right)\leq \Delta t_{\max}. \end{array}$$

In this formulation, θ denotes the learnable parameters of the Local Spatial Quality Head, β is the score-fusion weight, and a,b are the sigmoid calibration parameters. Here, $s^{\prime }\left(\theta ,\beta \right)={\left\{s_{i}^{\prime }\left(\theta ,\beta \right)\right\}}_{i=1}^{M}$, $s^{\prime \prime }\left(\theta ,\beta ,a,b\right)={\left\{s_{i}^{\prime \prime }\left(\theta ,\beta ,a,b\right)\right\}}_{i=1}^{M}$, and $y={\left\{y_{i}\right\}}_{i=1}^{M}$ denote the corresponding score and localization-quality vectors. ${\mathrm{AP}}_{\tau }$ denotes 3D average precision at IoU threshold τ, G denotes the ground-truth boxes, $\rho_{S}$ is Spearman’s rank correlation between the score and matched localization quality, QECE is the quality calibration error, and Δt is the additional inference-time cost introduced by the reliability layer. The mapping M(θ,β,a,b) is not optimized directly as a single differentiable objective during training. Instead, it summarizes the criteria used to design and evaluate the scoring layer. The ranking-oriented score vector $s^{\prime }\left(\theta ,\beta \right)$ is used for AP and Spearman’s rank correlation, whereas the calibrated score vector $s^{\prime \prime }\left(\theta ,\beta ,a,b\right)$ is used for Q-ECE because Q-ECE evaluates numerical score–quality alignment after calibration. The constraint ${\hat{b}}_{i}=b_{i}$ indicates that SAQC does not modify the decoded box geometry, and $\Delta t\left(\theta \right)\leq \Delta t_{\max}$ represents the real-time feasibility constraint. In the implementation, the local quality estimator is trained with a differentiable quality-regression loss, the fusion weight β is selected empirically, and the calibration mapping is learned with a soft-IoU negative log-likelihood, as described in the following subsections.

### 3.3. Baseline Detector and Score–Quality Inconsistency

The proposed framework is implemented on an SFA3D-style lightweight center-based BEV LiDAR detector, which is used as a controlled testbed for assessing score reliability with respect to localization quality in 3D vehicle detection [35]. Given a three-channel BEV map, the detector extracts multi-scale features using a ResNet-18 backbone and an FPN-based neck with KFPN fusion. Then it predicts object centers and box parameters via several task-specific heads, including heatmaps, center offsets, object direction, vertical position, and box dimensions. During inference, local maxima in the heatmap are decoded as object centers, and the associated regression outputs are used to construct the final 3D bounding boxes. The corresponding heatmap response is taken as the raw detection score *s* in this case.

Although this design is efficient for BEV-based detection, the raw score does not explicitly represent the localization quality of the decoded box. The heatmap branch is trained primarily to indicate object presence at the center location, whereas the regression branches are optimized to estimate geometric attributes. As a result, a detection may still receive a relatively high score even when its decoded box is not well aligned with the matched object. In this setting, the score can remain useful for classification-style ranking while still being imperfectly aligned with localization quality. For the present study, this mismatch is the key issue of interest. The proposed method does not seek to alter the decoded 3D box geometry produced by the baseline detector. Instead, it aims to improve the relationship between detector score and localization quality by introducing an additional quality-estimation branch based on local BEV spatial evidence. Figure 1 illustrates this issue directly in the baseline detector. Figure 1a shows the joint distribution of raw heatmap scores and matched BEV IoU for all detections. Although many high-quality detections are concentrated in the upper-right region, there is also a noticeable set of detections with relatively high scores but low IoU, indicating overconfident yet poorly localized predictions. Figure 1b further shows that the mismatch between score and localization quality becomes more pronounced at longer sensing ranges. These observations suggest that confidence reliability in BEV detection is spatially heterogeneous and motivate the proposed local spatial quality estimation strategy.

### 3.4. Local Spatial Quality Head

To improve score reliability with respect to localization quality, we introduce a Local Spatial Quality Head (LSQH) that estimates each detection’s localization quality from its local BEV feature neighborhood. The central idea is that the reliability of a decoded box is not determined solely by the detector response at a single center location. Instead, it is also influenced by the surrounding BEV structure, including local occupancy patterns, object extent, and nearby spatial arrangement. The proposed branch is therefore designed not to detect objects again, but to estimate localization quality from local spatial evidence and to support a more reliability-aware detection score. This design is particularly suitable for 3D vehicle detection in BEV LiDAR data because vehicles are rigid objects with relatively stable footprint, size, and orientation patterns in the horizontal plane. Their localization quality is therefore closely related to whether the decoded box is spatially consistent with the local BEV occupancy structure and vehicle extent. The specialization considered in this study is based on this BEV geometric regularity rather than on vehicle mobility; the proposed framework is a single-frame scoring method and does not use temporal motion cues.

Let $F\in R^{64\times H\times W}$ denote the intermediate BEV feature map bev_feat, and let $u_{i}=\left(r_{i},c_{i}\right)$ denote the center location of the *i*-th detection. For each detected center, a local feature patch is cropped from *F* as

(3) $$P_{i}=C_{k}\left(F,u_{i}\right),$$

where $C_{k}\left(\cdot ,\cdot \right)$ denotes the local BEV patch-sampling function with patch size *k*. In this study, k=7, and $P_{i}$ is a three-dimensional feature tensor with $\mathrm{shape}\left(P_{i}\right)=\left(64,7,7\right)$, corresponding to 64 feature channels and a 7×7 local BEV neighborhood. For $u_{i}=\left(r_{i},c_{i}\right)$, $C_{k}\left(F,u_{i}\right)$ samples the k×k neighborhood centered at $\left(r_{i},c_{i}\right)$, with boundary locations handled by zero padding. To explicitly encode the relative spatial arrangement within the local neighborhood, two coordinate channels are added to the cropped patch:

(4) $${\tilde{P}}_{i}=\Phi_{\mathrm{cat}}\left(P_{i},C\right)=\left[P_{i};C\right],$$

where $\Phi_{\mathrm{cat}}\left(\cdot ,\cdot \right)$ denotes channel-wise concatenation, and *C* is a two-channel coordinate tensor with shape shape(C)=(2,7,7), encoding the relative row and column offsets within the local patch. After concatenation, the augmented patch ${\tilde{P}}_{i}$ has tensor shape $\mathrm{shape}\left({\tilde{P}}_{i}\right)=\left(66,7,7\right)$, consisting of 64 BEV feature channels and 2 relative-coordinate channels. It is then used as the input to the quality head, allowing the branch to distinguish not only what local BEV evidence is present, but also where it is located relative to the detection center.

The augmented patch is processed by a lightweight convolutional network composed of two 3×3 convolutional layers with ReLU activations, followed by average pooling and a fully connected output layer. The quality head predicts a scalar quality estimate:

(5) $$q_{i}=f_{\theta }\left({\tilde{P}}_{i}\right),\;q_{i}\in \left[0,1\right],$$

where $f_{\theta }\left(\cdot \right)$ denotes the LSQH with learnable parameters θ. In this study, $q_{i}$ is interpreted as a localization-quality estimate derived from local BEV evidence, rather than as an additional classification confidence. In other words, the purpose of LSQH is not to replace the detector score, but to provide a spatially grounded quality signal for subsequent score fusion and reliability modeling.

An important feature of this design is that LSQH is attached to the detector as a lightweight auxiliary branch, without changing the base detector’s decoding process. The 3D box geometry remains unchanged, and the predicted quality score is used only for subsequent score fusion and calibration. In this sense, LSQH acts as a spatially informed reliability layer on top of the BEV detector, rather than a geometric refinement module.

### 3.5. Quality Supervision and Training

The LSQH is trained to predict the localization quality of each detected object using supervision derived from the base detector’s decoded output. Since the purpose of the proposed framework is to estimate geometric reliability rather than object presence, the supervision target is defined on positive matched training samples rather than on all locations. For each positive training sample, the detector predicts the box-related regression quantities at the corresponding location, including center offset, direction, vertical position, and box dimensions. These outputs are decoded into a 3D bounding box in the LiDAR coordinate system. Let ${\hat{b}}_{i}$ denote the decoded box for the *i*-th positive sample and $b_{i}^{*}$ denote its matched ground-truth box. The quality target $y_{i}\in \left[0,1\right]$ is defined by the following BEV IoU function:

(6) $$y_{i}=I_{\mathrm{BEV}}\left({\hat{b}}_{i},b_{i}^{*}\right)=\frac{\mathrm{area}\left(\pi_{\mathrm{BEV}}\left({\hat{b}}_{i}\right)\cap \pi_{\mathrm{BEV}}\left(b_{i}^{*}\right)\right)}{\mathrm{area}\left(\pi_{\mathrm{BEV}}\left({\hat{b}}_{i}\right)\cup \pi_{\mathrm{BEV}}\left(b_{i}^{*}\right)\right)},$$

where $I_{\mathrm{BEV}}\left(\cdot ,\cdot \right)$ denotes the BEV IoU function, and $\pi_{\mathrm{BEV}}\left(\cdot \right)$ projects a 3D bounding box onto its BEV footprint.

Since the proposed LSQH operates on local BEV feature patches and aims to improve score ordering with respect to localization quality in the BEV plane, using matched BEV IoU can provide a direct and geometrically meaningful supervision signal for the intended quality estimation task. Given the predicted quality score $q_{i}$ from the LSQH, the quality estimation loss is defined using the Smooth L1 function $\ell_{\delta }\left(\cdot \right)$:

(7) $$L_{q}=\frac{1}{N}\sum_{i=1}^{N}\ell_{\delta }\left(q_{i}-y_{i}\right),$$

where $\ell_{\delta }\left(\cdot \right)$ is defined as

(8) $$\ell_{\delta }\left(e\right)=\left\{\begin{array}{cc} \frac{1}{2}e^{2}/\delta , & |e|<\delta ,\\ \left|e\right|-\frac{1}{2}\delta , & \mathrm{otherwise}. \end{array}\right.$$

Here, *N* is the number of positive samples in the batch. This loss encourages the predicted quality score to approximate the matched BEV IoU while remaining stable during optimization. The total training objective combines the original detection loss of the base detector with the proposed quality loss:

(9) $$L=L_{\det}+\lambda_{q}L_{q},$$

where $L_{\det}$ denotes the original backbone detection loss and $\lambda_{q}$ controls the contribution of the quality supervision term.

Training for this framework follows a two-stage strategy to reduce feature-extractor distortion while allowing the quality branch to adapt to the detector representation. Let $\theta_{d}$ denote the parameters of the base detector and shared BEV feature extractor, and let $\theta_{q}$ denote the parameters of the LSQH. If the quality loss is applied to all parameters from the beginning, the auxiliary localization-quality objective may alter the shared BEV representation before the quality head has learned a stable mapping from local features to localization quality. Such premature feature drift may interfere with the detector’s original classification and regression behavior.

(10) $$\theta_{q}^{\left(1\right)}=\arg\min_{\theta_{q}}L_{q}\left(\theta_{q};\theta_{d}^{\left(0\right)}\right),\;\theta_{d}^{\left(0\right)}\;\mathrm{fixed}.$$

In the first stage, the detector backbone and feature layers are therefore frozen, and only the LSQH is optimized according to Equation (10). This stage makes the quality branch learn from a fixed BEV feature representation and prevents the auxiliary quality loss from immediately reshaping the shared feature extractor. In the second stage, all parameters are fine-tuned with the combined detection and quality objective in Equation (11) with a lower learning rate:

(11) $$\min_{\theta_{d},\theta_{q}}L_{\det}\left(\theta_{d}\right)+\lambda_{q}L_{q}\left(\theta_{d},\theta_{q}\right).$$

The detection loss $L_{\det}$ continues to regularize the shared representation during this stage, while the lower learning rate limits abrupt changes to the feature extractor. Thus, the second stage allows mild feature adaptation for localization-quality estimation without turning the quality branch into a dominant objective that distorts the base detector. Empirically, the resulting AP@0.7 remains close to the baseline while AP@0.8 and score–quality monotonicity improve, suggesting that the training strategy preserves the detector’s basic localization behavior while improving score ordering.

Through this supervision and training design, the predicted output of LSQH is explicitly encouraged to reflect localization quality derived from decoded geometry. The learned quality estimate then provides the basis for the score-fusion and calibration procedure described in the next subsection.

### 3.6. Quality-Aware Score Fusion and Post Hoc Quality Calibration

After local spatial quality prediction, the estimated localization quality is combined with the raw detector score to obtain a score that better reflects the reliability of each decoded detection. Let $s_{i}\in \left[0,1\right]$ denote the raw score of the *i*-th detection and let $q_{i}\in \left[0,1\right]$ denote the predicted quality score from the LSQH. Then, the fused score is defined as

(12) $$s_{i}^{\prime }=s_{i}\cdot q_{i}^{\beta },$$

where β>0 controls the contribution of the predicted quality term. Because the raw detector score already provides an initial ranking signal, the fusion is designed to preserve this baseline evidence while suppressing detections whose predicted localization quality is not supported by local spatial context. A larger β imposes a stronger penalty on detections with low predicted quality while β=0 reduces the formulation to the original detector score. The multiplicative fusion has two practical advantages. First, it is lightweight and requires no modifications to the decoded 3D box geometry or the base detector’s regression pipeline. Second, it naturally promotes detections that are simultaneously supported by both the original detector’s confidence and the estimated localization quality. As a result, the fused score $s_{i}^{\prime }$ is treated as a quality-aware ranking score, rather than as a calibrated probability-like output.

However, improving ranking consistency does not necessarily imply that the numerical scale of the fused score is well calibrated to localization quality. Specifically, the multiplicative interaction between $s_{i}$ and $q_{i}$ changes the score distribution and may shift it away from the scale required for calibration analysis. We therefore apply a final post hoc sigmoid calibration step to map the fused score to a calibrated quality score. Let ${\bar{s}}_{i}^{\prime }=\mathrm{clip}\left(s_{i}^{\prime },\epsilon ,1-\epsilon \right)$ denote the clipped fused score for numerical stability. The calibrated score is defined as

(13) $$s_{i}^{\prime \prime }=\sigma \left(a\cdot \mathrm{logit}({\bar{s}}_{i}^{\prime })+b\right),$$

where σ(·) denotes the sigmoid function, while *a* and *b* are scalar calibration parameters. In this study, calibration is performed with soft IoU targets [36] rather than binary correctness labels. Specifically, let $y_{i}\in \left[0,1\right]$ denote the matched BEV IoU, which is defined in Section 3.5. The calibration parameters are obtained by minimizing the soft negative log-likelihood:

(14) $$L_{\mathrm{cal}}=-\frac{1}{M}\sum_{i=1}^{M}\left[y_{i}\log s_{i}^{\prime \prime }+\left(1-y_{i}\right)\log\left(1-s_{i}^{\prime \prime }\right)\right],$$

where *M* is the number of detections used for calibration. Under this formulation, the calibrated output $s_{i}^{\prime \prime }$ is interpreted as a calibrated quality score, that is, a score whose numerical value is encouraged to match the expected localization quality, rather than a probability of categorical correctness.

The soft-IoU calibration objective in Equation (14) can be interpreted as a continuous-quality version of binary cross-entropy. Unlike a hard correctness label, the target $y_{i}\in \left[0,1\right]$ contributes fractional positive and negative terms according to the matched localization quality. Numerical stability is supported by three components. First, the clipping operation ${\bar{s}}_{i}^{\prime }=\mathrm{clip}\left(s_{i}^{\prime },\epsilon ,1-\epsilon \right)$ prevents the logit and logarithmic terms from becoming ill-conditioned when the fused score is close to 0 or 1. Second, the sigmoid parameterization maps the calibrated output back to (0,1), keeping $s_{i}^{\prime \prime }$ on the same bounded scale as the matched IoU target. Third, if $z_{i}=a\cdot \mathrm{logit}\left({\bar{s}}_{i}^{\prime }\right)+b$ and $s_{i}^{\prime \prime }=\sigma \left(z_{i}\right)$, the derivative of the per-sample soft negative log-likelihood with respect to $z_{i}$ is

(15) $$\frac{\partial L_{\mathrm{cal},i}}{\partial z_{i}}=s_{i}^{\prime \prime }-y_{i}.$$

Because both $s_{i}^{\prime \prime }$ and $y_{i}$ are bounded in [0,1], the calibration update is driven by a bounded residual between the calibrated score and the soft localization-quality target.

To empirically validate the calibration behavior while avoiding optimistic bias, the calibration analysis in this study adopts a 3-fold cross-fitted protocol on the validation set. The detections are partitioned into three folds. In each round, the calibration parameters (a,b) are estimated on two folds and then applied to the held-out fold. The out-of-fold calibrated scores from all three rounds are finally aggregated for quality calibration evaluation. This procedure prevents the calibration mapping from being evaluated on the same detections used to fit it, thereby providing a more reliable empirical assessment of the soft-IoU calibration formulation.

The proposed framework separates two related but distinct objectives through this design. The fusion step produces the quality-aware ranking score $s_{i}^{\prime }$ which is intended to improve score ordering with respect to localization quality. The post hoc calibration step then maps this score to the calibrated quality score $s_{i}^{\prime \prime }$ which is intended to improve the numerical agreement between score and matched localization quality.

## 4. Experiments

This section evaluates the proposed SAQC framework from detection performance, score–quality reliability, calibration quality, runtime efficiency, and ablation perspectives. We first describe the dataset, baseline detector, evaluation metrics, and implementation protocol. We then report overall detection performance, contextual comparisons with representative BEV LiDAR detectors, score reliability and calibration analyses, distance-wise behavior, and ablation results. These experiments are designed to determine whether SAQC improves score–localization reliability while keeping the decoded 3D box geometry unchanged.

### 4.1. Experimental Setup

The proposed framework was evaluated on the KITTI 3D object detection benchmark [37]. Experiments were conducted on the Car category using the commonly adopted split of 3769 training frames and 3712 validation frames. Detection performance was reported across the standard KITTI difficulty levels of Easy, Moderate, and Hard.

The main detection metric was 3D average precision (3D AP). Because the proposed method operates only on the scoring layer and does not introduce an explicit box-refinement step, any change in AP should be attributed to improved score ordering rather than geometric refinement. In addition to the conventional KITTI setting at IoU = 0.7, we also evaluated performance at a stricter threshold of IoU = 0.8. The stricter setting is particularly relevant in the present study because it is more sensitive to localization quality and therefore better reflects the ranking behavior targeted by the proposed framework. For example, in the KITTI Car 3D detection setting, predicted boxes are sorted by their confidence scores and matched to ground-truth car boxes. A predicted car box is counted as a true positive only when its 3D IoU with a matched ground-truth box satisfies the specified threshold. As a simple numerical example, a detection with 3D IoU =0.76 would satisfy an IoU =0.7 criterion but fail an IoU =0.8 criterion. This illustrates why the stricter threshold used in this study is more sensitive to whether high-scoring detections are also well localized.

The base detector was an SFA3D-style lightweight center-based BEV LiDAR detector with a relatively simple and transparent prediction pipeline. We use this detector as a controlled testbed rather than as a new detection backbone or a state-of-the-art leaderboard detector. Its heatmap-based scoring, dense BEV feature representation, and direct center-based box decoding make the score–localization relationship easier to isolate. This is important because the goal of the present study is to evaluate the effect of a scoring-layer reliability module, rather than to compare complete detector architectures with different refinement, fusion, or post-processing designs [35]. This detector is therefore a suitable baseline for the present study because the effect of quality-aware rescoring can be examined without substantial interference from more complex refinement or fusion modules. Training followed the two-stage strategy described in Section 3.5: the detector feature extractor was first frozen while the Local Spatial Quality Head was optimized, and all parameters were then jointly fine-tuned using a smaller learning rate.

In addition to 3D AP, several score–quality metrics were used to evaluate the proposed framework. The monotonic relationship between score and localization quality was measured using Spearman’s rank correlation coefficient between the detection score and the matched BEV IoU [38]. Post hoc quality calibration was evaluated using the quality calibration error (Q-ECE), defined as the difference between the mean predicted score and the mean matched BEV IoU within each score bin. Correspondingly, the reliability plots reported in this study are quality reliability diagrams, in which the horizontal axis represents the mean predicted score and the vertical axis represents the mean matched BEV IoU. This formulation evaluates continuous quality calibration rather than binary correctness calibration.

The selected metrics correspond to the reliability-oriented goal of this study. 3D AP is retained as the standard object-detection metric, and it is score-sensitive because detections are ranked by confidence before AP is computed. Spearman’s rank correlation coefficient assesses whether the final score yields a monotonic ordering consistent with the matched localization quality. This is important because SAQC is designed to improve score ordering rather than to modify decoded box geometry. Q-ECE evaluates a different property: whether the numerical score value agrees with the expected matched BEV IoU after binning. Therefore, Spearman’s ρ measures ranking reliability, whereas Q-ECE measures numerical calibration quality. The mean score is reported only to document the score-scale shift caused by multiplicative fusion, and the mean IoU of retained detections indicates whether retained detections are better localized on average. Runtime metrics are reported to quantify the computational cost of the additional reliability layer, not as evidence of detection accuracy.

During inference, the predicted quality score was fused with the raw detector score according to Section 3.6 with β∈{0.5, 1.0, 1.5, 2.0}. A final post hoc calibration step was then applied to the fused score. To avoid fitting and evaluating the calibration mapping on the same samples, post hoc calibration was assessed using a 3-fold cross-fitted protocol on the validation set. In each round, the calibration parameters were estimated in two folds and applied to the held-out fold. The out-of-fold predictions from all three rounds were aggregated for final evaluation. Temperature scaling and Platt-style sigmoid calibration with soft IoU targets were both included for comparison.

To further examine the source of the gain, the proposed method was compared with several ablation variants. These included a point-wise quality head using only the 1×1 center feature, a spatial patch variant without relative coordinate channels, and models with different local patch sizes. These settings were designed to test whether the observed improvements arise specifically from local BEV neighborhood evidence and explicit spatial encoding, rather than from simply adding an auxiliary quality-prediction branch. This ablation design also provides a controlled quantitative comparison with scalar quality-estimation designs inspired by prior quality-aware scoring work. The point-wise quality head uses only the center feature and represents a quality branch without local spatial context, whereas the spatial and coordinate-aware variants test whether local BEV neighborhood evidence provides additional benefit under the same detector, dataset split, and evaluation protocol.

Overall, the experiments were designed to evaluate the proposed framework from four complementary perspectives: detection performance, score–quality monotonicity, post hoc quality calibration, and the contribution of local spatial context.

### 4.2. Overall Detection Performance

Table 1 reports the 3D AP of the proposed SAQC framework under different fusion weights β on the KITTI Car validation set. Across all tested fusion weights, SAQC improves over the baseline, with more pronounced gains under the stricter IoU threshold of 0.8. Because the proposed method does not modify the decoded 3D box geometry, these improvements should be interpreted as the effect of better score ordering rather than geometric refinement. Importantly, the AP results in Table 1 are computed using the fused SAQC ranking score $s^{\prime }$, before post hoc sigmoid calibration. The post hoc calibration parameters are not used to compute the AP values in Table 1; they are only applied in the post hoc quality calibration analysis reported in Section 4.3. Therefore, the observed AP gains, especially under IoU = 0.8, should be attributed to quality-aware score fusion and improved ranking of better localized detections, rather than to biased calibration parameters. This behavior is consistent with the intended role of SAQC as a reliability-oriented scoring layer.

For the Moderate setting, 3D AP@0.7 increases from 89.13 in the baseline to 89.40, 89.50, 89.57 and 89.60 for β=0.5, 1.0, 1.5 and 2.0, respectively. Under the stricter IoU threshold of 0.8, the corresponding AP improves from 74.40 to 75.39, 75.57, 75.72 and 75.78. A similar trend can be observed for the Hard setting, where 3D AP@0.7 rises from 89.35 to 89.59, 89.63, 89.67 and 89.69, while AP@0.8 improves from 75.41 to 76.15, 76.29, 76.42 and 76.40. Two observations are particularly important. First, the absolute gains at IoU = 0.7 are relatively modest. This is expected because the baseline detector already provides strong performance under the conventional evaluation threshold. Second, the gains at IoU = 0.8 are consistently larger in both Moderate and Hard settings. These results indicate that the proposed reliability-oriented rescoring mechanism preferentially promotes detections with better localization quality, and that this effect becomes more visible under stricter overlap criteria. These AP results also provide a sanity check for the two-stage training strategy. Although AP does not directly measure feature-extractor distortion, the fact that AP@0.7 remains at least comparable to the baseline suggests that the auxiliary quality branch does not cause an observable degradation in the detector’s standard evaluation setting. The larger gains at AP@0.8 further indicate that the main effect is improved ranking of better localized detections under stricter overlap criteria.

**Figure 3:**
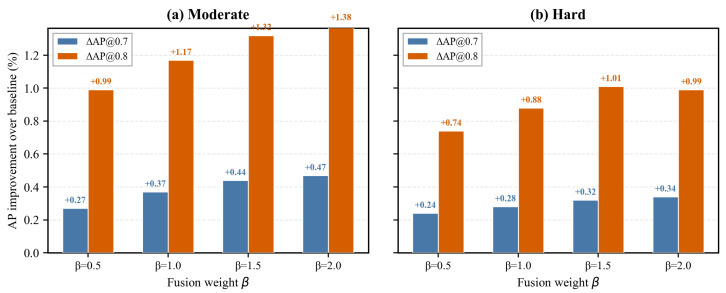
AP improvement over the SFA3D baseline under different fusion weights β. (**a**) Moderate and (**b**) Hard results evaluated at IoU thresholds of 0.7 and 0.8.

Figure 3 further highlights this trend by showing the AP improvements over the baseline for the Moderate and Hard setting. In both cases, the gain under IoU = 0.8 is substantially larger than that under IoU = 0.7 across all tested values of fusion weight β. For example, in the Moderate setting, the AP improvement at IoU = 0.8 reaches +1.32 for β=1.5 and +1.38 for β=2.0, whereas the corresponding gains at IoU = 0.7 are +0.44 and 0.47. In the Hard setting, the largest gains at IoU = 0.8 are +1.01 for β=1.5 and +0.99 for β=2.0. Among the tested configurations, β=1.5 and β=2.0 provide the strongest overall AP performance. Since the subsequent experiment also considers score–quality consistency and calibration behavior in addition to AP, β=1.5 is adopted as the default setting for the following sections. Overall, these results suggest that increasing the contribution of the predicted quality score is beneficial up to a point, after which the AP gain becomes marginal.

To place the selected baseline in a broader BEV LiDAR detection context, we further evaluated representative detectors on the same KITTI Car training/validation split using the same 3D AP evaluation protocol. The comparison includes PointPillars, a pillar-based BEV detector, and CenterPoint, a center-based detector, together with the SFA3D baseline and SFA3D + SAQC. This comparison is intended to contextualize the selected baseline and the SAQC-enhanced model rather than to serve as a state-of-the-art leaderboard comparison.

As shown in Table 2, the selected SFA3D baseline is within the performance range of representative BEV LiDAR detectors under the same validation setting. The AP differences among PointPillars, CenterPoint, and SFA3D mainly reflect differences in complete detector architectures and training designs. In contrast, SAQC is evaluated as a reliability-oriented scoring layer on top of a fixed detector. Therefore, the relevant controlled comparison remains between SFA3D and SFA3D + SAQC, with the detector architecture and decoded box geometry kept unchanged.

**Figure 4:**
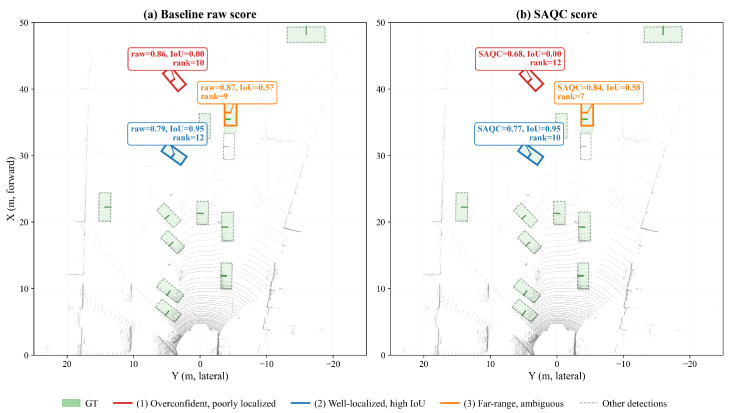
Qualitative KITTI validation example illustrating the effect of SAQC rescoring. The left panel shows detections ranked by the baseline raw score, and the right panel shows matched detections ranked by the fused SAQC ranking score $s^{\prime }$. Highlighted detections show an overconfident low-IoU case (1), a well-localized high-IoU case (2), and an ambiguous far-range case (3).

Figure 4 provides a qualitative KITTI validation example to illustrate how SAQC changes detection scores and ranks. This example is not intended as additional quantitative evidence, but as a visual explanation of the score reliability behavior reported in Table 1. The left panel shows detections ranked by the baseline raw score, whereas the right panel shows matched detections ranked by the fused SAQC ranking score $s^{\prime }$. In this frame, SAQC lowers the rank of an overconfident detection with low matched IoU, preserves a well-localized high-IoU detection, and adjusts the rank of an ambiguous far-range detection. Because the proposed scoring layer does not introduce an explicit box-refinement step, the example should be interpreted as illustrating score and rank changes rather than geometric correction.

In addition to detection accuracy, we measured the inference speed of the baseline detector and the proposed SAQC framework in Table 3. This analysis is included to quantify the deployment cost of the proposed reliability layer, not to serve as evidence of improved detection accuracy. Since SAQC adds local patch extraction and quality-head evaluation after the base detector, the relevant question is whether the improvement in score reliability is obtained while preserving real-time operation. The results show that SAQC increases mean latency from 6.38 ms to 9.65 ms, but the full system still reaches 103.6 FPS on an NVIDIA GeForce RTX 4070 Ti GPU (NVIDIA, Santa Clara, CA, USA). Thus, Table 3 should be interpreted as a speed–reliability trade-off analysis: the method introduces measurable overhead, but remains within a real-time operating regime.

### 4.3. Score Reliability and Post Hoc Calibration Analysis

Beyond AP, we further examine whether SAQC improves score reliability with respect to matched localization quality and how post hoc calibration affects the numerical agreement between score and quality. Table 4 summarizes the pre-calibration score–quality behavior under different fusion weights β.

Compared with the baseline detector, quality-aware rescoring consistently strengthens the monotonic relationship between detection score and matched BEV IoU. Spearman’s rank correlation coefficient increases from 0.3685 in the baseline to 0.3949, 0.4019, 0.4043 and 0.4016 for different fusion weights. At the same time, the mean matched IoU of retained detections also increases from 0.868 to 0.874, 0.876, 0.878, and 0.879 for β=0.5, 1.0, 1.5, and 2.0. These results indicate that quality-aware rescoring improves ranking reliability with respect to localization quality, with β=1.5 providing the best overall trade-off before post hoc calibration.

Table 4 also shows that the pre-calibration Q-ECE increases as β becomes larger. This trend is expected and is not a contradiction to the improvement of the ranking. As discussed in Section 3.6, the fused score $s^{\prime }$ is designed as a quality-aware ranking score rather than as a numerically calibrated quality value. The multiplicative fusion step redistributes the score scale to better reflect localization quality, but this new scale is not guaranteed to remain calibrated before post hoc correction.

**Figure 5:**
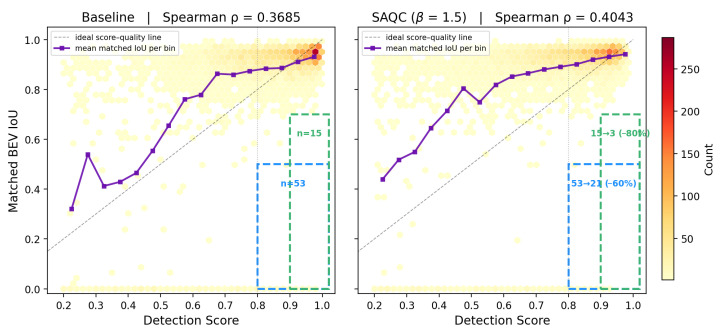
Comparison of the score–IoU relationship between the baseline detector and the proposed quality-aware rescoring framework (β=1.5).

Figure 5 provides a direct visualization of this effect for the baseline detector and the default SAQC setting (β=1.5). For the proposed method, the mean matched-IoU curve in the high-score region aligns more closely with the ideal score–quality line, suggesting better agreement between confidence and localization quality. Instances with score >0.8 and IoU <0.5 decrease from 53 to 21, and those with score >0.9 and IoU <0.7 fall from 15 to 3. Around scores 0.5–0.6, the proposed method shows a slight dip in mean matched-IoU, which mainly reflects the downward remapping of previously overconfident detections rather than a loss of detection capability. Overall, these observations support the interpretation that SAQC selectively suppresses poorly localized detections while preserving high scores for better localized ones.

To evaluate the numerical agreement between score and localization quality after post hoc correction, Table 5 reports the 3-fold cross-fitted post hoc quality calibration results for the baseline detector and the default SAQC setting (β=1.5). Temperature scaling provides only limited correction in this setting, whereas the proposed Platt-style sigmoid calibration with soft IoU targets produces a substantially lower Q-ECE. For the baseline detector, Q-ECE decreases from 0.0632 to 0.0284 after sigmoid calibration. For SAQC (β=1.5), the corresponding value decreases from 0.0995 to 0.0183. Compared with the calibrated baseline, the calibrated SAQC score achieves a 35.6% lower Q-ECE. These results indicate that although the fused score is not well calibrated numerically before post hoc correction, it becomes better aligned with localization quality after the calibration mapping is applied. The pre-calibration Q-ECE values in Table 5 are recomputed within the 3-fold cross-fitted evaluation protocol for consistency with the calibration analysis and are therefore not numerically identical to the whole-validation pre-calibration values reported in Table 4.

**Figure 6:**
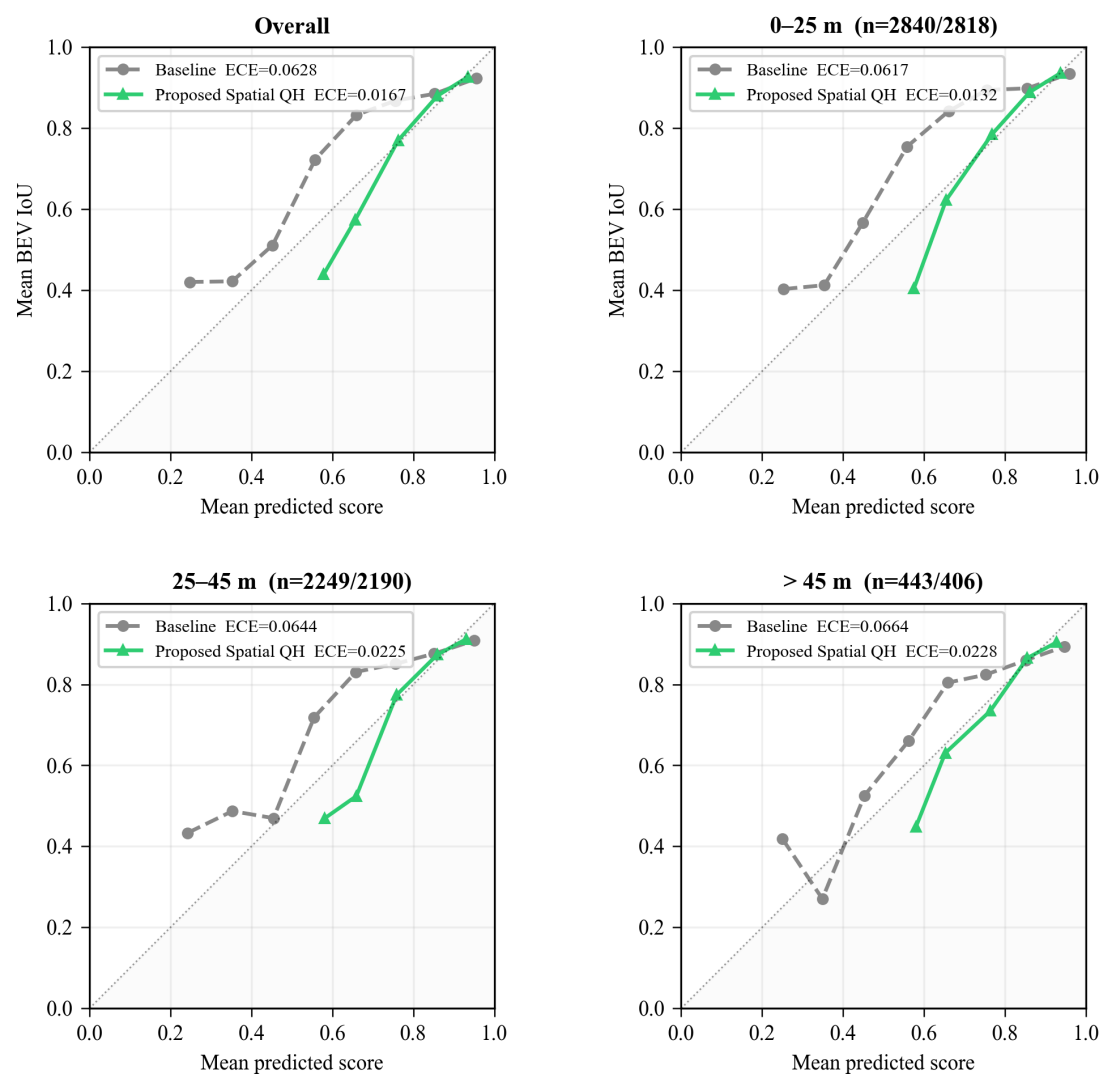
Quality reliability diagrams for the raw baseline heatmap score and the final Platt-calibrated SAQC score (β=1.5) on the full validation set and across different distance ranges.

Figure 6 further demonstrates the quality-reliability diagrams for the raw baseline heatmap score and the final Platt-calibrated SAQC score across the full validation set and across different distance ranges. This comparison visualizes the reliability change from the original detector output to the final SAQC output after quality-aware rescoring and post hoc calibration. The calibrated SAQC score tracks the matched BEV IoU more closely across most score bins, with the clearest improvement in the low- to mid-score region where the baseline tends to overestimate localization quality. The benefit remains visible across sensing ranges, including the far-range regime, where sparse point-cloud observations make localization more ambiguous.

Taken together, the results in Table 4 and Table 5 and Figure 5 and Figure 6 support a consistent interpretation of SAQC: the fusion step improves score structure for ranking reliability, whereas the subsequent post hoc calibration step improves the numerical agreement between scores and matched quality.

### 4.4. Bootstrap Stability of Reliability Metrics

To assess the statistical stability of the reliability improvements, we conducted a paired scene-level bootstrap with 1000 resamples on the KITTI validation split, as shown in Table 6. In each replicate, validation frames were sampled with replacement, and the same sampled frame set was used for both the baseline and SAQC. We then recomputed score–quality monotonicity and post hoc quality calibration metrics and reported percentile confidence intervals for the paired differences.

SAQC improved Spearman’s rank correlation between detection score and matched BEV IoU by 0.036, with a 95% percentile confidence interval of [0.023, 0.048]. For post hoc quality calibration, SAQC reduced Q-ECE by 0.0098 after sigmoid calibration, with a 95% confidence interval of [−0.0141, −0.0054]. Since both intervals exclude zero, the bootstrap results indicate that the improvements in score–quality monotonicity and calibrated quality alignment are stable under validation-scene resampling.

### 4.5. Ablation Study

To further examine whether the observed gains come from local BEV spatial evidence rather than from adding a generic quality estimator, we conducted a controlled comparison under the same SFA3D-style detector, KITTI split, and evaluation protocol. In addition to the baseline detector, we include two adapted quality-aware baselines motivated by prior LiDAR quality-estimation studies. The first is an IoU-aware Scalar QH, inspired by IoU-aware confidence estimation methods such as 3D IoU-Net and CIA-SSD [28,29], which predicts localization quality from the 1×1 center feature without using local BEV context. The second is an LMD-style Metadata QH, inspired by lightweight post-processing quality-estimation methods for LiDAR detectors [30], which predicts localization quality from detection-level metadata rather than local BEV feature patches. These adapted baselines are not intended as full reimplementations of the original detector architectures; instead, they isolate whether the proposed coordinate-aware local BEV evidence provides additional benefit over scalar or metadata-based quality estimation. Table 7 further includes a Patch QH w/o Coord. variant and the full SAQC model to separate the effects of local BEV context and relative coordinate encoding.

The results show that simply attaching an auxiliary scalar quality branch is not sufficient. The IoU-aware Scalar QH, which represents a quality-estimation design without local BEV context, yields a very low post-calibration Q-ECE. However, it does not improve score–quality monotonicity and slightly reduces AP@0.7 in the Moderate and Hard settings relative to the baseline. This indicates that a scalar quality signal based only on the center feature may be numerically easy to calibrate after post hoc correction, but it is insufficient for improving ranking reliability with respect to localization quality.

The LMD-style Metadata QH provides a post-processing quality-estimation baseline that uses detection-level metadata rather than local BEV features. Its performance indicates whether range, box geometry, raw score, and point-count-related cues can explain localization quality without directly using the local feature neighborhood. Compared with this metadata-based baseline, the patch-based variants test whether local BEV feature evidence provides additional spatial support for score–localization reliability.

When the quality head is given a 7×7 local patch without coordinate channels, the behavior changes noticeably. Compared with the IoU-aware Scalar QH, the Patch QH w/o Coord. improves score–quality monotonicity from 0.3630 to 0.3821 and recovers AP@0.7 performance, suggesting that local BEV neighborhood context provides useful evidence beyond the center feature alone. However, local context without explicit coordinate encoding does not provide the strongest strict-IoU performance, indicating that spatial evidence must also be represented in a position-aware manner.

The full SAQC model achieves the best overall trade-off among the compared variants. With both local patch context and relative coordinate encoding, SAQC improves Moderate AP@0.7 from 88.94 to 89.57, Moderate AP@0.8 from 75.58 to 75.72, and Spearman’s ρ from 0.3630 to 0.4043 compared with the IoU-aware Scalar QH. Compared with the LMD-style Metadata QH, SAQC improves Moderate AP@0.7 from 89.22 to 89.57, Hard AP@0.7 from 89.31 to 89.67, Moderate AP@0.8 from 75.46 to 75.72, and Spearman’s ρ from 0.3790 to 0.4043, while slightly reducing post-calibration Q-ECE from 0.0195 to 0.0183. These controlled comparisons support the claim that the gain comes from coordinate-aware local BEV spatial reliability modeling rather than from adding a generic auxiliary or post-processing quality estimator alone.

Table 8 further examines the effect of patch size with coordinate channels. As the local patch increases from 1×1 to 7×7, 3D AP@0.8, Spearman’s ρ, and post-calibration Q-ECE all improve progressively. In particular, the score–quality monotonicity rises from approximately 0.3725 at 1×1 to 0.4043 at 7×7, while post-calibration Q-ECE decreases from 0.0252 to 0.0183. This trend indicates that localization-quality estimation benefits from a moderate local neighborhood that captures object extent and nearby spatial structure. It is worth noting that the 1×1 configuration in Table 8 is not identical to the point-wise quality head in Table 7. The former includes explicit relative coordinate channels, whereas the latter relies solely on the center feature. As a result, although the point-wise variant achieves a lower post-calibration error, it does not improve score–quality monotonicity or strict-threshold detection performance. In contrast, the coordinate-aware variants introduce a more structured score distribution that better reflects spatial relationships, thereby improving ranking behavior but making numerical calibration slightly more challenging. This observation further supports the view that the goal of SAQC is not to minimize calibration error alone, but to achieve balanced improvements in ranking reliability, detection performance, and post-calibration quality alignment.

Increasing the patch size further to 9×9 does not provide a meaningful additional gain. The performance remains close to that of the 7×7 setting, but the improvement becomes marginal while the number of additional parameters increases. This phenomenon suggests that overly large local regions introduce diminishing returns, likely because they incorporate more redundant context than useful localization evidence. Therefore, the 7×7 configuration provides the most favorable operating point in the present study.

Taken together, the ablation results support the central claim of this study: localization quality in BEV detection is spatially structured, and improving score reliability depends on modeling informative local spatial evidence around the detected object.

## 5. Discussion

The results show that the proposed SAQC framework improves BEV LiDAR 3D vehicle detection primarily through score restructuring rather than geometric refinement. Because the decoded 3D box geometry remains unchanged, the observed gains in 3D average precision should be interpreted as improvements in score reliability with respect to localization quality, especially under the stricter IoU threshold of 0.8. This distinction highlights an often underemphasized aspect of detector performance: beyond generating accurate boxes, a practically useful LiDAR perception system should also assign scores that meaningfully prioritize better-localized predictions.

A key observation of this study is that ranking reliability and numerical calibration should be treated as related but distinct properties. The experimental results show that quality-aware fusion improves the monotonic relationship between detection score and matched localization quality, effectively suppressing overconfident but poorly localized detections. However, this improvement in ranking behavior does not directly translate into well-calibrated scores. Instead, the fused score should be interpreted as a quality-aware ranking signal. At the same time, a separate post hoc calibration step is required to align the score’s numerical scale with localization quality. This separation clarifies the complementary roles of score restructuring and calibration in reliability modeling. The scene-level bootstrap analysis further supports this interpretation: the improvements in Spearman’s rank correlation and post-calibration Q-ECE remain stable under validation-scene resampling, indicating that the reliability gains are not driven by a small subset of scenes.

More importantly, the ablation results provide evidence that localization reliability in BEV detection is intrinsically spatially structured. A point-wise quality head based solely on the center feature can produce a score that is easier to calibrate numerically. Still, it does not improve score–quality monotonicity or strict-IoU detection performance. In contrast, incorporating local BEV neighborhood context consistently improves ranking reliability, and explicit relative-coordinate encoding further enhances this effect. The patch-size analysis shows that performance improves progressively as the local neighborhood expands to a moderate size, after which the gain becomes marginal. These findings suggest that informative local spatial context—rather than isolated feature responses—is the key factor for estimating localization quality in BEV LiDAR detection.

From a LiDAR sensing perspective, this behavior is practically relevant because detector outputs are often used as object-level spatial measurements in downstream perception, tracking, planning, and decision-making pipelines. In such settings, the usefulness of a detector depends not only on detection accuracy but also on whether higher scores correspond to more reliable spatial estimates. The proposed framework increases the mean matched IoU of retained detections, reduces the number of high-score but poorly localized cases, and maintains consistent reliability improvements across sensing ranges, including more ambiguous far-range scenarios. These results suggest that a spatially informed scoring mechanism may improve the interpretability and practical usability of BEV LiDAR detection outputs in real-time sensing applications.

The runtime analysis further shows that the proposed reliability layer introduces a measurable but acceptable inference overhead. Although SAQC adds only a small number of learnable parameters, the inference latency increases because local feature patches must be extracted around detected centers and passed through the quality head. Nevertheless, the full system remains real-time, achieving 103.6 FPS on an RTX 4070 Ti. This result indicates that SAQC improves score reliability while preserving the baseline BEV detector’s lightweight operating regime.

This study has several limitations. First, the current validation is conducted on a single SFA3D-style lightweight center-based BEV detector and a single dataset, KITTI. The comparison with quality-aware baselines is conducted within the same detector rather than as a full cross-detector benchmark comparison across different LiDAR detector backbones. This controlled design isolates the effect of the proposed scoring layer, but it also limits claims about direct transfer to stronger detectors with multi-stage refinement, transformer-based feature aggregation, or multi-sensor fusion. In its current form, SAQC assumes a dense BEV feature map aligned with decoded detection centers, so applying it to point-based, proposal-based, range-view, or anchor-based detectors would require redefining how local spatial evidence is associated with each detection. Future work should evaluate SAQC on additional center-based BEV detector families and compare quality-aware scoring methods under a unified experimental protocol. Second, the experiments are limited to the KITTI Car category. Vehicles provide a suitable first target for the proposed local spatial reliability model because they have relatively stable BEV footprints and rigid geometry. However, this does not imply that the mechanism is specific to vehicle mobility. The current framework does not use temporal motion information, and its effectiveness for smaller, more deformable, or more irregular object classes such as pedestrians and cyclists remains to be examined. Third, the supervision target is based on BEV IoU, which is well aligned with the method’s design but does not capture all aspects of full 3D geometric accuracy. Fourth, although the proposed method remains real-time, the runtime overhead indicates that patch extraction and quality-head evaluation should be further optimized for deployment in more resource-constrained sensor platforms. Future work may extend the framework to other detector architectures, additional object classes, more diverse LiDAR datasets, and alternative quality targets that integrate BEV and full 3D localization criteria. Fifth, the present study does not explicitly model the physical propagation of LiDAR signals through the atmosphere. In real sensing environments, molecular scattering and absorption, aerosols, fog droplets, rain, snow, and humidity-related particle growth can attenuate LiDAR returns, introduce backscatter, reduce effective point density, and increase localization uncertainty [39,40,41]. These effects are important for LiDAR sensing reliability, but they are outside the direct modeling scope of the proposed SAQC framework. SAQC operates after point-cloud acquisition and detector decoding; it estimates whether the final detector score is well aligned with the localization quality of the decoded box. Therefore, the reported improvements should be interpreted as improvements in detector-output reliability rather than as evidence of atmospheric robustness or physical ranging correction. Future work should evaluate the proposed reliability modeling strategy on adverse-weather LiDAR datasets, controlled weather-chamber measurements, or physically degraded point clouds to examine whether local BEV reliability cues remain effective under explicit attenuation and backscatter conditions.

## 6. Conclusions

This study examined a specific reliability problem in BEV LiDAR 3D vehicle detection: the final detector score may not reliably indicate how well the decoded 3D box is localized. To address this problem, we proposed SAQC, a lightweight scoring layer that estimates localization quality from a coordinate-augmented local BEV feature patch and fuses this estimate with the raw detector score. The method does not change the decoded 3D box coordinates; it changes how detections are ranked and calibrated.

On the KITTI Car validation split, SAQC improved Moderate 3D AP from 89.13 to 89.57 at IoU =0.7, and from 74.40 to 75.72 at the stricter IoU =0.8 setting when β=1.5. The score–IoU Spearman correlation increased from 0.3685 to 0.4043, and the post-calibration Q-ECE decreased from 0.0284 for the calibrated baseline to 0.0183 for calibrated SAQC. These results show that the main benefit is better score ordering and score–quality alignment, rather than geometric box refinement.

The ablation study further showed that this improvement does not come merely from adding an auxiliary quality head. Local BEV neighborhood context and relative coordinate encoding were both important for improving strict-IoU AP and score–quality monotonicity. Runtime evaluation showed that the full system still achieved 103.6 FPS on an RTX 4070 Ti, indicating that the reliability layer remains compatible with real-time BEV LiDAR perception.

The current validation is limited to one SFA3D-style detector, one dataset, and the Car category, and it does not directly model atmospheric attenuation, backscatter, or adverse-weather degradation. Future work should evaluate the method across additional detector families, object classes, datasets, and physically degraded LiDAR conditions.

## Figures and Tables

**Table 1:** Overall detection performance of the proposed framework under different quality fusion weights β on the KITTI dataset. The symbol “—” indicates that the fusion weight is not applicable to the baseline detector.

Method	β	AP@0.7 Easy	AP@0.7 Mod.	AP@0.7 Hard	AP@0.8 Easy	AP@0.8 Mod.	AP@0.8 Hard
Baseline (SFA3D)	—	97.09	89.13	89.35	83.58	74.40	75.41
SAQC	0.5	97.80	89.40	89.59	84.20	75.39	76.15
SAQC	1.0	97.73	89.50	89.63	84.48	75.57	76.29
SAQC	1.5	97.97	89.57	89.67	84.71	75.72	76.42
SAQC	2.0	97.87	89.60	89.69	84.82	75.78	76.40

**Table 2:** Comparison with representative BEV LiDAR 3D detectors on the KITTI Car validation split.

Method	Description	3D AP@0.7		
		Easy	Moderate	Hard
PointPillars [10]	Pillar-based BEV	98.49	91.31	90.24
CenterPoint [12]	Center-based BEV	97.54	88.29	88.61
SFA3D [35]	Lightweight center-based BEV	97.09	89.13	89.35
SFA3D + SAQC	Reliability layer on SFA3D	97.97	89.57	89.67

**Table 3:** Runtime comparison between the baseline detector and the proposed SAQC framework.

Method	Extra Params	FPS	Mean Latency	Median	P95
SFA3D baseline	–	156.7	6.38 ms	6.20 ms	8.41 ms
SFA3D + SAQC	+12.9 K (+0.1%)	103.6	9.65 ms	9.44 ms	12.21 ms

**Table 4:** Score–quality monotonicity and pre-calibration behavior under different fusion weights β. The symbol “—” indicates that the fusion weight is not applicable to the baseline detector.

Method	β	Spearman’s ρ	Pre-Calibration Q-ECE	Mean Score	Mean IoU of Retained Detections
Baseline (SFA3D)	—	0.3685	0.0628	0.835	0.868
SAQC	0.5	0.3949	0.0775	0.811	0.874
SAQC	1.0	0.4019	0.0879	0.794	0.876
SAQC	1.5	0.4043	0.0991	0.779	0.878
SAQC	2.0	0.4016	0.1136	0.766	0.879

**Table 5:** 3-fold cross-fitted post hoc quality calibration results for the baseline detector and the proposed SAQC framework (β=1.5).

Method	Pre-Calibration Q-ECE	Q-ECE + Temperature Scaling	Q-ECE + Sigmoid Calibration
Baseline	0.0632	0.0652	0.0284
SAQC (β=1.5)	0.0995	0.0839	0.0183

**Table 6:** Paired scene-level bootstrap confidence intervals for reliability metrics. Differences are reported as SAQC minus baseline.

Metric	Δ Mean	95% Percentile CI	CI Excludes 0
Spearman’s ρ	+0.036	[+0.023, +0.048]	Yes
Q-ECE after sigmoid calibration	−0.0098	[−0.0141, −0.0054]	Yes

**Table 7:** Controlled comparison of quality-aware baseline variants and the proposed SAQC framework.

Method	3D AP@0.7 Mod.	3D AP@0.7 Hard	3D AP@0.8 Mod.	Spearman’s ρ	Post-Cal. Q-ECE
Baseline	89.13	89.35	74.40	0.3685	0.0284
IoU-aware Scalar QH	88.94	88.69	75.58	0.3630	0.0092
LMD-style Metadata QH	89.22	89.31	75.46	0.3790	0.0195
Patch-based QH w/o Coord.	89.35	89.48	75.50	0.3821	0.0237
Proposed SAQC	89.57	89.67	75.72	0.4043	0.0183

**Table 8:** Effect of local patch size on detection performance, score–quality monotonicity, and post-calibration quality calibration.

Patch Size	Additional Parameters	3D AP@0.7 (Mod.)	3D AP@0.7 (Hard)	3D AP@0.8 (Mod.)	Spearman’s ρ	Post-Calibration Q-ECE
1×1	0.26 K	89.05	89.10	75.20	0.3725	0.0252
3×3	2.4 K	89.25	89.38	75.35	0.3823	0.0234
5×5	6.6 K	89.45	89.55	75.58	0.3951	0.0209
7×7	12.9 K	89.57	89.67	75.72	0.4043	0.0183
9×9	21.4 K	89.52	89.60	75.62	0.3994	0.0196

## Data Availability

The data used in this study are publicly available from the KITTI 3D object detection benchmark. Additional processed data and code are available from the corresponding author upon reasonable request.
